# Intramuscular injection of human umbilical cord-derived mesenchymal stem cells improves cardiac function in dilated cardiomyopathy rats

**DOI:** 10.1186/s13287-017-0472-y

**Published:** 2017-01-28

**Authors:** Chenggang Mao, Xu Hou, Benzhen Wang, Jingwei Chi, Yanjie Jiang, Caining Zhang, Zipu Li

**Affiliations:** 1 Department of Pediatrics, the Affiliated Hospital of Qingdao University, 16 Jiangsu Rd, Qingdao, 266003 China; 2 Department of Endocrinology, the Affiliated Hospital of Qingdao University, Qingdao, China; 3 The Institute of Metabolic Diseases, Qingdao University, Qingdao, China; 4 Department of Pediatrics, Qingdao Women and Children’s Hospital, 6 Tongfu Rd, Qingdao, 266011 China

**Keywords:** Mesenchymal stem cells (MSCs), Dilated cardiomyopathy (DCM), Intramuscular injection, Cardiac function, Paracrine

## Abstract

**Background:**

Stem cells provide a promising candidate for the treatment of the fatal pediatric dilated cardiomyopathy (DCM). This study aimed to investigate the effects of intramuscular injection of human umbilical cord-derived mesenchymal stem cells (hUCMSCs) on the cardiac function of a DCM rat model.

**Methods:**

A DCM model was established by intraperitoneal injections of doxorubicin in Sprague-Dawley rats. hUCMSCs at different concentrations or cultured medium were injected via limb skeletal muscles, with blank medium injected as the control. The rats were monitored for 4 weeks, meanwhile BNP, cTNI, VEGF, HGF, GM-CSF, and LIF in the peripheral blood were examined by ELISA, and cardiac function was monitored by echocardiography (Echo-CG). Finally, the expression of IGF-1, HGF, and VEGF in the myocardium was examined by histoimmunochemistry and real-time PCR, and the ultrastructure of the myocardium was examined by electron microscopy.

**Results:**

Injection of hUCMSCs markedly improved cardiac function in the DCM rats by significantly elevating left ventricular ejection fraction (LVEF) and left ventricular fraction shortening (LVFS). The BNP and cTNI levels in the peripheral blood were reduced by hUCMSCs, while HGF, LIF, GM-CSF, and VEGF were increased by hUCMSCs. Expression of IGF-1, HGF, and VEGF in the myocardium from the DCM rats was significantly increased by hUCMSC injection. Furthermore, hUCMSCs protected the ultrastructure of cardiomyocytes by attenuating mitochondrial swelling and maintaining sarcolemma integrity.

**Conclusions:**

Intramuscular injection of UCMSCs can improve DCM-induced cardiac function impairment and protect the myocardium. These effects may be mediated by regulation of relevant cytokines in serum and the myocardium.

**Electronic supplementary material:**

The online version of this article (doi:10.1186/s13287-017-0472-y) contains supplementary material, which is available to authorized users.

## Background

Dilated cardiomyopathy (DCM) is a condition characterized by enlargement of the left ventricular chamber associated with systolic dysfunction, without thickening of the left ventricular wall [1]. The clinical presentation of DCM includes progressive heart failure, arrhythmia, thromboembolism, and myocardial infarction [1]. Based on epidemiological data derived from different countries [2], the incidence of DCM is approximately 80/100,000. DCM accounts for nearly 50% of pediatric cardiomyopathies [3]. Although genetic defects, infection-induced myocarditis, neuromuscular disorders, and congenital metabolic disorders have been suggested as contributing factors for the pathogenesis of DCM [4], the underlying pathogenic mechanisms of DCM still remains evasive currently.

As a refractory life-threatening pediatric condition which eventually leads to heart failure, heart transplantation remains the ultimate treatment for DCM [5]. Due to the invasive and costly nature of heart transplantation, alternative therapies with higher accessibility are imperative for pediatric DCM. Mesenchymal stem cells (MSCs), which boast self-renewal and multipotency capacities, can promote angiogenesis, tissue repair and immunoregulation via paracrine mechanisms [6], therefore providing a potential therapeutic strategy for pediatric DCM [7]. Currently, the role of stem cells in attenuating pediatric DCM remains poorly understood. A recent study reported that intramyocardial injection of human umbilical cord-derived MSCs (hUCMSCs) could attenuate ventricular remodeling by inhibiting cardiomyocyte apoptosis, promoting angiogenesis and differentiation of cardiac progenitor cells [8]. On the other hand, many studies have shown evidence that MSCs secrete a number of cytokines via paracrine mechanisms to protect cardiomyocytes from infarction-induced injuries [9].

Previous studies have reported that intracoronary [10] and intramyocardial [11] administration of autologous bone marrow-derived MSCs (BMMSCs) improve left ventricular ejection fraction (LVEF) and other cardiac function parameters. However, their application is restricted by the inherent invasiveness associated with the procedures. Intramyocardial injection-induced scar formation and calcification may trigger arrhythmia and microinfarction [12], while MSCs administered via intravascular injection accumulate at the lungs, liver and spleen instead of the heart [13]. On the other hand, studies by Shabbir et al. [14] showed that intramuscular injection of MSCs improved the cardiac function of a cardiomyopathy hamster model, while the grafted MSCs primarily remained in the muscles, suggesting intramuscular injection as a minimally invasive approach may be a feasible pathway of administration.

In the present study, we report that intramuscular injection of hUCMSCs improved the cardiac function in a doxorubicin-induced DCM rat model and attenuated DCM-associated mitochondria and sarcolemma impairments. Intraperitoneal injection of doxorubicin has been a long-established protocol for induction of DCM in animal models [15]. We have also found that hUCMSC injection elevated the myocardial expression and circulating levels of relevant cytokines including hepatocyte growth factor (HGF), insulin-like growth factor-1 (IGF-1), leukocyte inhibitory factor (LIF), granulocyte-macrophage colony-stimulating factor (GM-CSF), and vascular endothelial growth factor (VEGF), suggesting that paracrine mechanisms are likely to contribute to the therapeutic effects of hUCMSCs on DCM. This study has demonstrated that hUCMSCs can deliver therapeutic effects on DCM through the minimally invasive approach of intramuscular injection, thus significantly broadening the accessibility of stem cell therapy for pediatric DCM.

## Methods

### Establishment of the DCM model

Adult male Sprague-Dawley rats (average body weight 180 ± 20 g) purchased from Shandong Lukang Pharmaceutical (Jining, Shandong, China) were caged and maintained in an SPF-grade animal facility at the Affiliated Hospital of Qingdao University. The rats were randomly divided into two groups: the DCM group (n = 140) in which doxorubicin (2 mg per kg body weight) was injected intraperitoneally once a week for 8 consecutive weeks, and the normal control group (n = 20) in which saline (2 mL) was injected in the same manner. Development of DCM was examined and verified by echocardiography (Additional file 1: Figure S1) and the levels of brain natriuretic peptide (BNP) and troponin I (cTNI) in the peripheral blood derived from the caudal vein (Additional file 1: Table S1). The animals were maintained in a comfortable living environment including optimum caging, temperature, humidity and lighting with free access to water and food. All the procedures involving the rats were approved by the Ethics Committee in Animal Research of Qingdao University according to the university regulations for animal research.

During the procedure, the rats were monitored every 12 hours regarding their fur appearance, body temperature, body weight, ascites, breathing, and food intake. While all the rats in the normal control group behaved normally in their daily activities and grew at normal rate, 82 out of 140 rats (58.6%) in the DCM group survived at 2 weeks after the end of the procedure (Additional file 1: Figure S2). The surviving rats showed bad appetite, reduced movements, delayed reactions, slowed growth, hair loss, and ascites.

### hUCMSC preparation

Umbilical cords were collected from healthy newborn children immediately after their birth with the fully informed consent from their legal guardians and approval from the Shandong Provincial Medical Ethics Council. After screening for human immunodeficiency virus (HIV), hepatitis C virus (HCV), cytomegalovirus (CMV), hepatitis B virus (HBV) and syphilis spirochete, the Wharton’s jelly was mechanically separated from the umbilical cords and dissected into small pieces, which were then resuspended in the MesenCult-SF culture medium (Stemcell Technologies, Vancouver, BC, Canada) supplemented with 2 mM Glutamax (Invitrogen, Carlsbad, CA, USA), plated onto CellBIND™ cell culture dishes (Corning, Corning, NY, USA) and cultured in a humidified 5% CO_2_ atmosphere at 37 °C. The medium was replaced by half at a 3-day interval until the attached cells grew to 80% confluence. The cells were then detached with 0.05% trypsin and expanded for another passage before storage in liquid nitrogen. Prior to injection, the cells were resuscitated and screened for aerobes, mycoplasma, HIV, HBV, HCV, CMV, and the presence of endotoxins. The cells were also examined for growth capacity, differentiation capacity, and immune phenotype (CD34, CD44, CD45, CD90, CD105, and HLA-DR) (Additional file 1: Figure S3).

### hUCMSC treatment

The surviving DCM rats were randomly divided into four groups (n = 16 each): the DCM control group in which 2.0 mL MesenCult medium was injected, the supernatant group injected with 2.0 mL of conditioned hUCMSC culture medium, the low-dose group injected with 2.0 mL of hUCMSC suspension (2.5 × 10^5^ cells), and the high-dose group injected with 1.0 × 10^6^ hUCMSCs (2.0 mL). Meanwhile, the normal control rats were injected with 2.0 mL PBS.

At 2 weeks after the establishment of the DCM rat model, treatment was initiated via intramuscular injection into the forelimb skeletal muscle (0.3 mL each side) and hindlimb muscle (0.35 mL at two points on each side). The general condition of the rats was observed for 24 h after the injection for adverse events. A second treatment of the same regimen was delivered at 2 weeks after the initial treatment.

### Echocardiography

At immediately prior to the initial treatment and 2 weeks after the second treatment, the rats were anesthetized by intraperitoneal injection of Nembutal (30 mg per kg body weight) and their cardiac function examined via echocardiography using a Philips iE33 xMATRIX Echocardiography System (Philips Healthcare, Amsterdam, Netherland) equipped with a 8–12 MHz ultrasound probe. The left ventricular long-axis view and the apical four-chamber view were examined by two-dimensional and M-mode echocardiography. A number of cardiac function-related parameters were determined, including left ventricular end-diastolic diameter (LVEDd), left ventricular end-systolic diameter (LVESd), left ventricular posterior wall thickness (LVPWT), interventricular septum thickness (IVST), left ventricular ejection fraction (LVEF) and left ventricular fractional shortening (LVFS).

### ELISA

Inner canthus blood was extracted at immediately prior to the initial treatment and 2 weeks after the second treatment. BNP, cTNI, VEGF, HGF, GM-CSF, and LIF concentrations in the serum were determined by ELISA (ELISA kits were purchased from Senxiong Biotech, Shanghai, China). The assays were conducted according to the manual instructions.

### Histoimmunochemistry

At 2 weeks after the second treatment, the rats were euthanized by intraperitoneal injection of a lethal dose of Nembutal (200 mg per kg body weight). The heart was immediately dissected, fixed by 4% paraldehyde and then embedded in paraffin. Sections (5 μm in thickness) were dewaxed by successively soaking in xylene, anhydrous ethanol, 70% ethanol, and water. After treatment with 3% H_2_O_2_ for 30 min, the sections were soaked in 0.5% Triton X-100 for 30 min. The sections were then blocked for 30 min by 1% bovine serum albumin in PBS before incubated at 4 °C overnight with the primary antibodies diluted (at 1:100–500) in the blocking solution, followed by incubation with peroxidase-conjugated secondary antibodies (at 1:500–1000) at room temperature for 2 h and developed with DAB reagents. The sections were then counterstained with hematoxylin. After staining, the sections were dehydrated by successively soaking in water, 70% ethanol, anhydrous ethanol, and xylene. The sections were eventually mounted with Clearmount (Invitrogen) and examined by using an Olympus BX51 microscope (Olympus, Tokyo, Japan). The primary antibodies used in these assays included rabbit anti-rat HGF, rabbit anti-rat IGF-1 and rabbit anti-VEGF antibodies, all purchased from Abcam (Cambridge, MA, USA).

The obtained images were analyzed by the Image-Pro Plus 6.0 software (Media Cybernetics, Rockville, MD, USA). For each cytokine, five sections were analyzed and five positively stained areas were chosen from each section. The gray scale of the chosen areas was determined and the average gray scale was calculated as a quantitative index for protein expression.

### Real-time quantitative PCR

Heart tissues were collected immediately after euthanasia and total RNA was extracted from homogenized heart tissues using the TRI reagent (1 mL per 50–100 mg of tissue; Sigma-Aldrich). Chloroform was added to the RNA extract at a ratio of 1:5 (v/v). After mixing, phase separation was achieved by centrifugation at 12,000 g at 4 °C for 15 min. Isopropylalcohol (500 μL) was added to 500 μL of the aqueous phase. The samples were centrifuged again at 12,000 g at 4 °C for 10 min. RNA extract in the pellets were washed with 75% ethanol, and then resuspended in DEPC-H_2_O.

RT-PCR was performed at 37 °C for 60 min using a GoScript reverse transcription system (Promega, Madison, WI, USA) which employs an M-MLV Reverse Transcriptase to produce cDNA from the RNA extracts. Quantitative PCR on the derived cDNA products was performed using a SYBR Premix Ex Taq system (Takara Bio, Otsu, Japan) with a Roche LightCycler 96 real-time PCR system (Roche, Penzberg, Germany). Primers designed for HGF, IGF-1, and VEGF were synthesized by Invitrogen China (Shanghai, China; sequences shown in Table 1). The reaction mixture was composed of 4 μL PCR master mix (5×), 0.1 μL each of the forward and reverse primers, 0.5 μL cDNA template, 0.1 μL Ex Taq HS polymerase and 15.2 μL H_2_O. The quantitative PCR reactions consisted of 40 cycles of denaturation at 95 °C for 5 s, annealing at 60 °C for 20 s followed by extension at 72 °C for 1 min. GAPDH gene was used as the normalization control. The cycle number at fluorescence threshold (CT) was used as an indicator of the expression level of the target gene. The expression levels in the normal controls were used as the comparative reference, and relative quantification of the expression in the study groups was determined by 2^ΔΔCT^ values, in which ΔCT = CTtarget gene – CTGAPDH and ΔΔCT = ΔCT_study group_ – ΔCTnormal control.

**Table 1:** RT-PCR primer sequences

Target gene	Forward prime	Reverse prime
VEGF	GGTGTGGTCTTTCGTCCTTCT	GATGGGTTTGTCGTGTTTCTG
HGF	GGCCATGGTGCTACACTCTT	TGTGGGGGTACTGCGAATC
IGF-1	TCTACCTGGCACTCTGCTTG	CCTGTGGGCTTGTTGAAGTA

### Electron microscopy

Small pieces (approximately 1 mm^3^) of heart tissue were fixed with 2.5% glutaraldehyde in 0.1 M sodium cacodylate buffer at 4 °C overnight and then postfixed in 1% osmium tetroxide-1.5% potassium ferrocyanide for 1 h. After dehydration in ethanol-acetone, the tissues were embedded in Epon812 epoxy resin (E Micron Technologies, Shanghai, China). Ultrathin sections (50 nm-thick) were prepared using a Leica EM UC6 ultramicrotome (Leica, Wetzlar, Germany), and stained with uranyl acetate-lead citrate. Finally, ultrastructure of the heart tissues was examined by a JEOL JEM-1200EX transmission electron microscope (JEOL, Tokyo, Japan).

### Statistics

Statistical analyses were performed using the SPSS 13.0 software (SPSS Inc., Chicago, IL, USA). Data were expressed as mean ± SEM. One-way ANOVA in conjunction with least significant difference test was performed to determine statistical significance (*P* < 0.05).

## Results

### Effects of hUCMSCs on the survival of DCM rats

The survival rate of the supernatant group, low-dose group, high-dose group, and DCM control group was 81.25% (13/16), 87.50% (14/16), 81.25% (13/16), and 75.00% (12/16) respectively. Notably, the low-dose and high-dose hUCMSC treatments resulted in improvement in appetite and movements, and attenuation in hair loss and ascites, while no such improvements were observed in the other groups. No local inflammation, ulceration or anabrosis was detected at the injection sites. Autopsy of the dead rats during the course of hUCMSC treatment showed hepatomegaly, hepatonecrosis, sanguineous ascites, pleural effusion, renomegaly, and intestinal obstruction, suggesting that they were likely to die from complications of heart failure.

### Effects of hUCMSCs on cardiac function of DCM rats

**Fig. 1:**
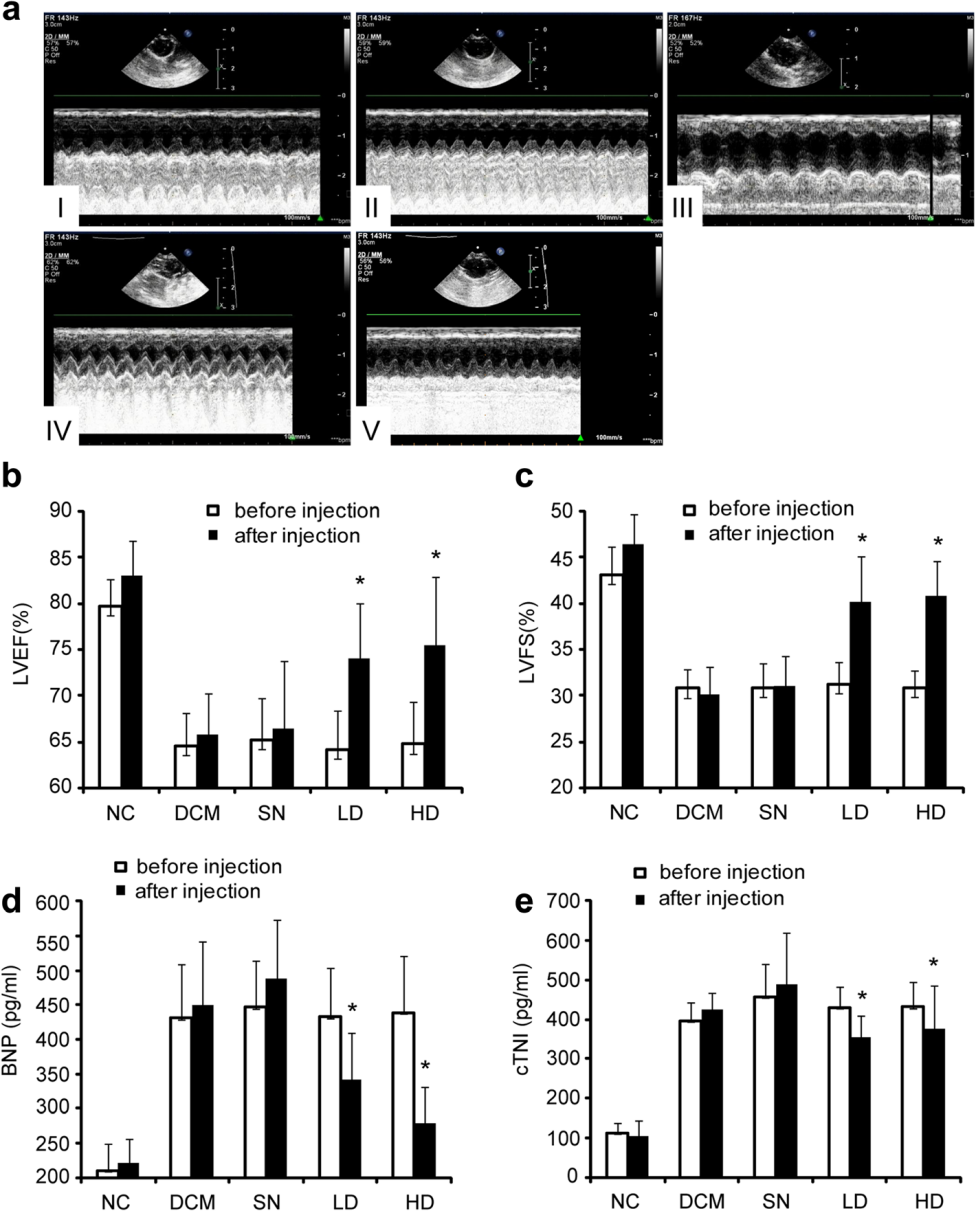
**a** Echocardiography of the normal control (*I*), DCM control (*II*), supernatant-treated (*III*), low-dose hUCMSC-treated (*IV*), and high-dose hUCMSC-treated (*V*) rats. **b**–**e** LVEF, LVFS, serum BNP, and serum cTNI of the normal control (NC), DCM control (DCM), supernatant-treated (SN), low-dose hUCMSC-treated (LD), and high-dose hUCMSC-treated (HD) rats. *Empty bars* represent LVEF before hUCMSC treatment and *black solid bars* for LVEF after hUCMSC treatment. *Asterisks* represent statistical difference (*P* < 0.05, ANOVA). *BNP* brain natriuretic peptide, *cTNI* troponin I, *LVFS* left ventricular fraction shortening, *LVEF* left ventricular ejection fraction

Heart dimensions (represented by LVIDd, LVESd, LVPWd, and IVST) and cardiac function (represented by LVEF and LVFS) were similar in all the study groups before hUCMSC treatment. Compared to before hUCMSC treatment, hUCMSC treatment did not result in significant changes in left ventricular dimensions (including LVIDd, LVESd, LVPWd, and IVST) in any of the study groups (Fig. 1a, detailed data not shown). On the other hand, at 2 weeks after the second hUCMSC treatment, significant elevation was observed in LVEF (Fig. 1b) and LVFS (Fig. 1c) in the low-dose, and high-dose hUCMSC groups (*P* < 0.05), while LVEF and LVFS remained constant after hUCMSC treatment in the DCM control and supernatant groups (*P* > 0.05, Fig. 1b–c). For the two hUCMSC doses used in this study, no dose-effect relationship was observed for hUCMSCs on cardiac function in terms of LVEF and LVFS (*P* > 0.05, Fig. 1b–c).

While all the study groups had similar serum BNP and cTNI levels before hUCMSC treatment (Fig. 1d–e), serum BNP (Fig. 1d) and cTNI (Fig. 1e) were markedly lowered after hUCMSC treatment in the low-dose and high-dose groups (*P* < 0.05). No significant alterations in serum BNP and cTNI levels were detected in the DCM control and supernatant group (*P* > 0.05). Again, no dose-effect relationship was present for hUCMSCs on cardiac function as reflected by serum BNP and cTNI levels (*P* > 0.05).

### Effects of hUCMSCs on cytokine levels in the peripheral blood of DCM rats

**Fig. 2:**
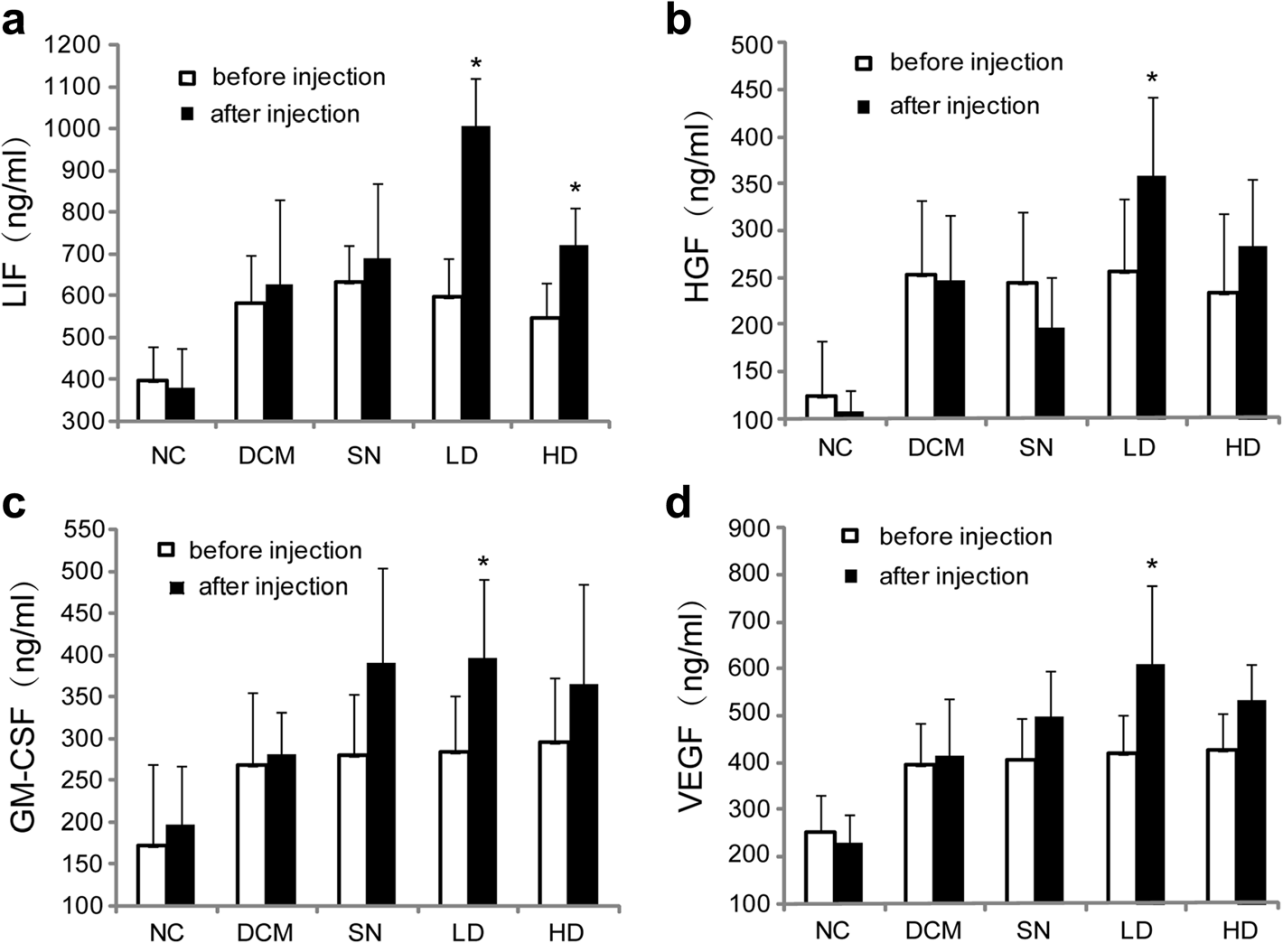
Serum concentrations of LIF (**a**), HGF (**b**), GM-CSF (**c**), and VEGF (**d**) in different groups. *Empty bars* represent LVEF before hUCMSC treatment and *black solid bars* for LVEF after hUCMSC treatment. *Asterisks* represent statistical difference compared with the DCM group (*P* < 0.05, ANOVA). *DCM* DCM control group, *GM-CSF* granulocyte-macrophage colony-stimulating factor, *HD* high-dose hUCMSC-treated group, *HGF* hepatocyte growth factor, *IGF-1* insulin-like growth factor-1, *LC* low-dose hUCMSC-treated group, *LIF* leukocyte inhibitory factor, *NC* normal control group, *SN* supernatant-treated group, *VEGF* vascular endothelial growth factor

While doxorubicin injection resulted in significant higher serum levels of HGF, LIF, GM-CSF, and VEGF as compared to the normal controls (*P* < 0.05), no difference was present in serum HGF, LIF, GM-CSF, and VEGF levels among the study groups. The low-dose hUCMSC treatment induced significant elevations in the serum levels of LIF, HGF, GM-CSF, and VEGF (*P* < 0.05, Fig. 2a–d), while the high-dose hUCMSC treatments led to a significant elevation in serum LIF level only (*P* < 0.05, Fig. 2a). The conditioned medium treatments did not induce significant alterations in the serum levels of the cytokines above (*P* > 0.05). Within the two doses of hUCMSCs used in this study, no dose-effect relationship was detected (*P* > 0.05).

### Effects of hUCMSCs on cytokine expression in the heart of DCM rats

**Fig 3:**
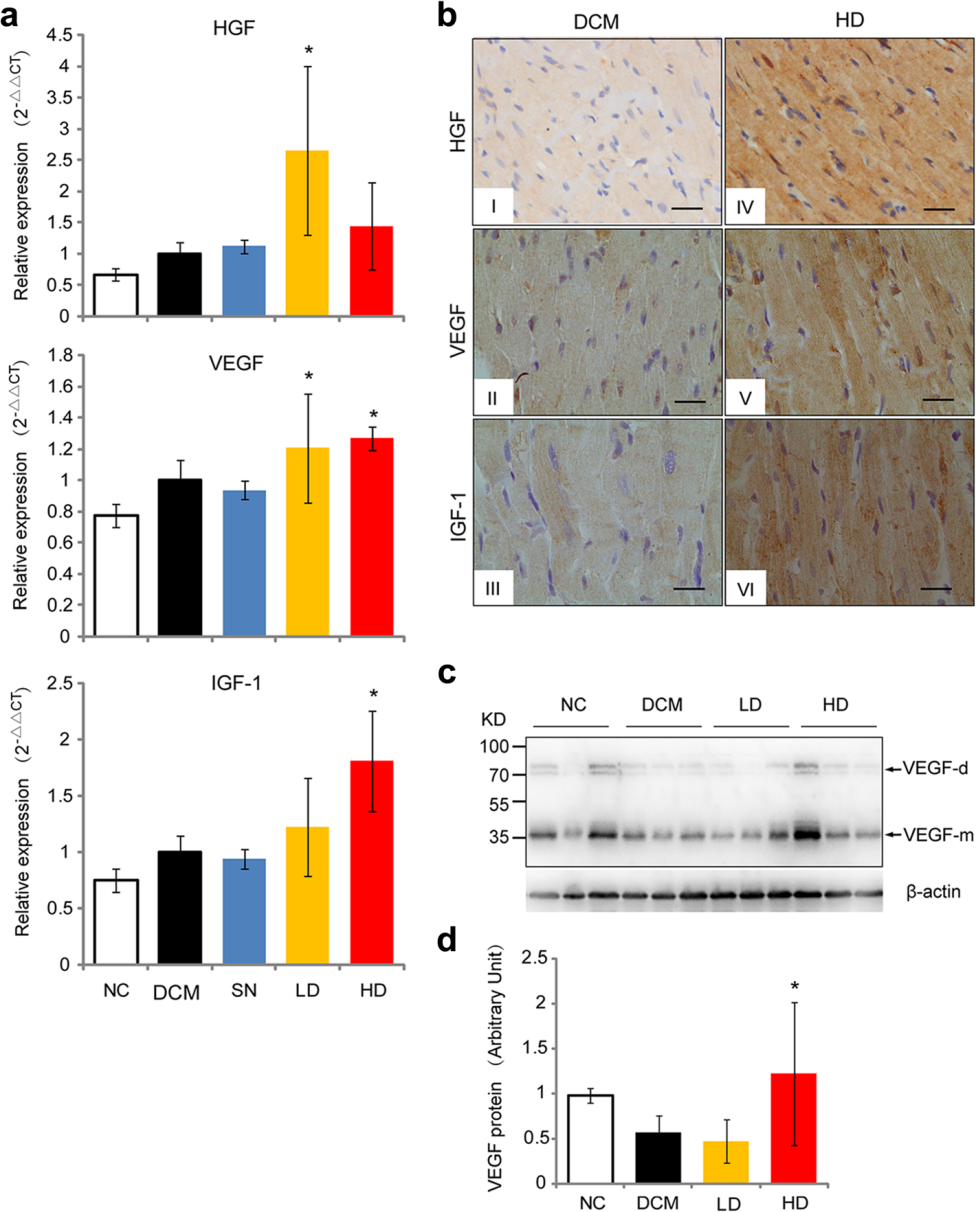
Expression of HGF, VEGF, and IGF-1 in the heart as determined by real-time quantitative PCR (**a**). *Asterisks* represent statistical difference compared with the DCM group (*P* < 0.05, ANOVA). **b** Histoimmunochemical stain of HGF (*I* and *IV*), VEGF (*II* and *V*), and IGF-1 (*III* and *VI*). Expression of these cytokines represented by yellow to brown stain was predominantly localized in the cytoplasm*. Left panels* (*I-III*) are heart tissues from the DCM control group, and *right panels* (*IV-VI*) from the high-dose group. *Black bars* = 50 μm. **c** Representative figure showing expression of VEGF in the heart detected by Western blot. β-actin was detected as a loading control. VEGF-m and VEGF-d represent VEGF monomer and dimer respectively. **d** Densitometric analysis of VEGF expression. *Asterisks* represent statistical difference compared with the DCM group (*P* < 0.05, ANOVA). *DCM* DCM control group, *HD* high-dose hUCMSC-treated group, *HGF* hepatocyte growth factor, *IGF-1* insulin-like growth factor-1, *LC* low-dose hUCMSC-treated group, *NC* normal control group, *SN* supernatant-treated group, *VEGF* vascular endothelial growth factor

According to the real-time quantitative PCR results, all the DCM rats showed elevated expression of HGF, VEGF, and IGF-1 compared to the normal control rats (*P* < 0.05, Fig. 3a). Compared with the DCM control group, a significant increase was observed in HGF and VEGF expression by the low-dose treatments (*P* < 0.05, Fig. 3a), while the high-dose treatments induced significant elevations in VEGF and IGF-1 expression (*P* < 0.05, Fig. 3a). On the other hand, treatment with the conditioned medium did not alter the expression of HGF, VEGF, or IGF-1 (*P* > 0.05).

Histoimmunochemical examination of heart tissues revealed that expression of HGF, VEGF, and IGF-1 was predominantly localized in the cytoplasm (Fig. 3c), and seemed to be increased in high-dose hUCMSC-treatment groups. The expression of VEGF was detected using Western blot. The results showed that VEGF expression was significantly increased in the high-dose group compared with the DCM control group (*P* < 0.05, Fig. 3d).

### Effects of hUCMSCs on cardiomyocyte structure in DCM rats

**Fig. 4:**
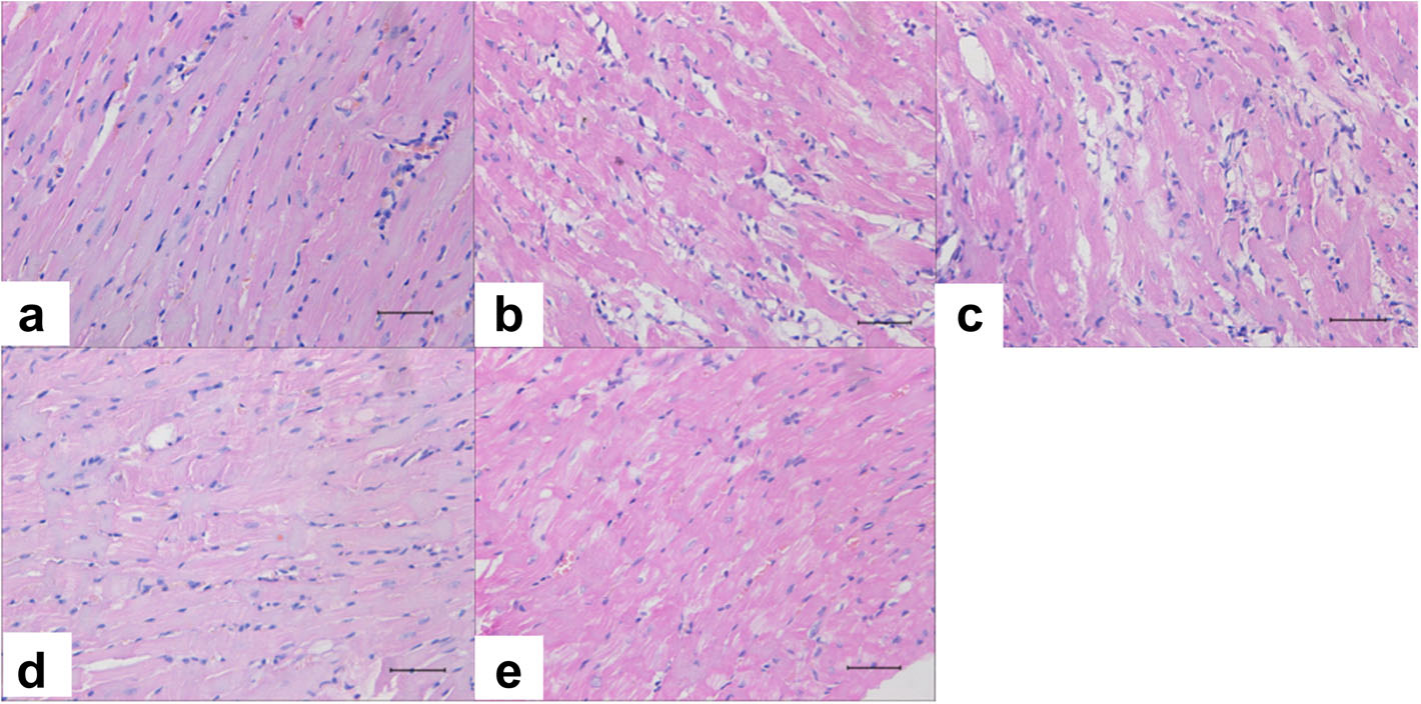
Hemoxylin-eosin stain of heart tissues derived from the normal control (**a**), the DCM control (**b**), the supernatant group (**c**), the low-dose group (**d**), and the high-dose group (**e**). *Black bars* = 50 μm

Hemoxylin-eosin stain of the heart from the normal control rats showed that myocardial fibers were orderly arranged and the nuclei uniformly stained with light tinges, absent of necrosis, interstitial hemorrhage, or infiltration of inflammatory cells (Fig. 4a). In contrast, heart tissues from the DCM control and supernatant groups showed chaotic arrangement of myocardial fibers with some fiber ruptures, extensive dropsy, and vacuolar degeneration of cardiomyocytes, and interstitial dropsy with mild infiltration of inflammatory cells (Fig. 4b and c). The cardiomyocyte impairments listed above were markedly attenuated in the low-dose and high-dose groups (Fig. 4d–e).

Cardiac fibrosis was evaluated by Masson staining. Meanwhile, the collagen volume fraction (CVF = area of the collagen/area of field of vision × 100%) was measured according to Masson staining. Masson staining of the DCM group showed a large number of green-stained collagen deposition among the myocardial fibers, not only around blood vessels (Additional file 1: Figure S4 AII). In the low-dose (Additional file 1: Figure S4 AIII) and high-dose group (Additional file 1: Figure S4 AIV), it showed significantly reduced fibrous tissue deposition compared to the DCM group. CVF was also significantly reduced in the low-dose and high-dose group compared to the DCM group (*P* < 0.05) (Additional file 1: Figure S4 B).

**Fig. 5:**
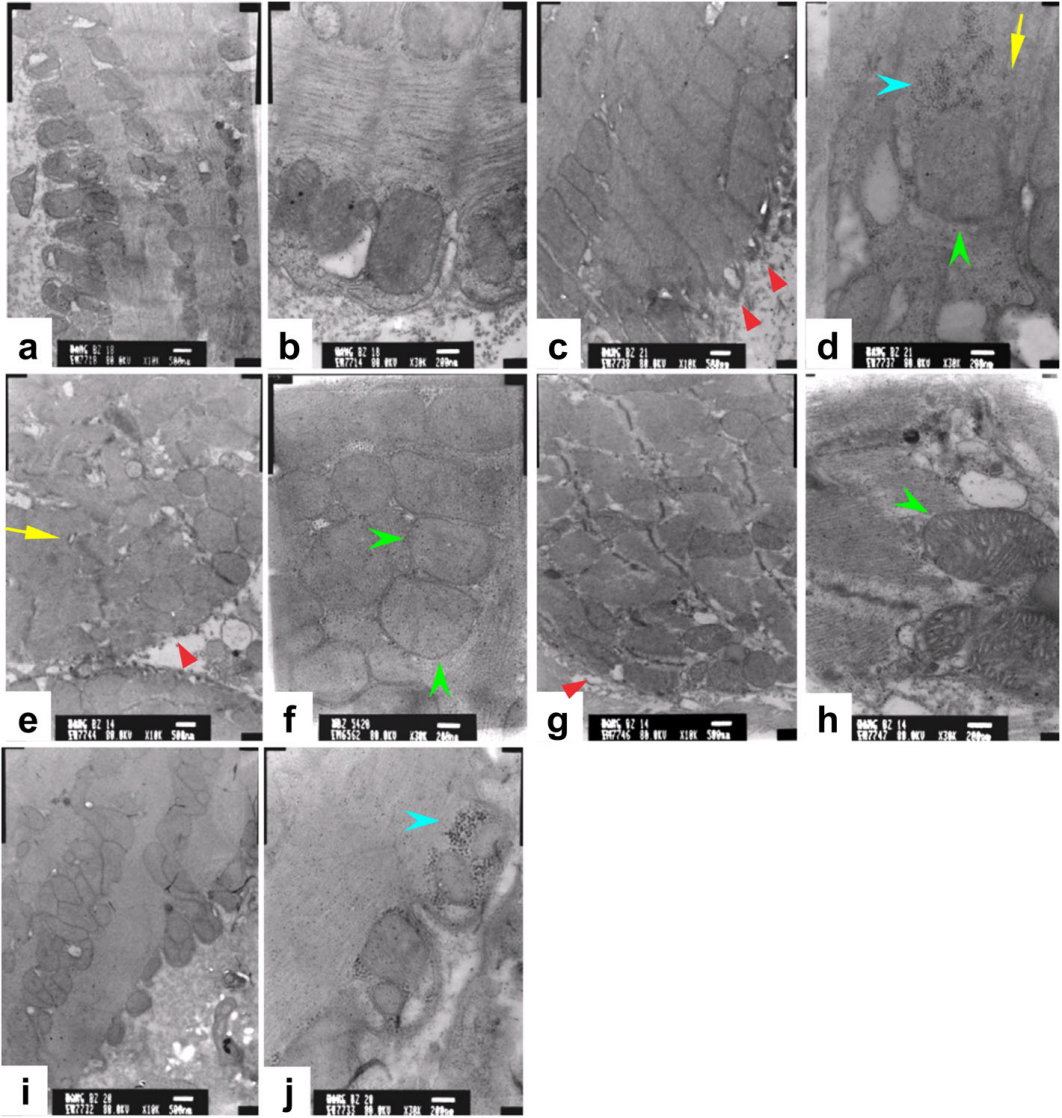
Transmission electron microscopy images of heart tissues derived from the normal control (**a**–**b**), the DCM control (**c**–**d**), the supernatant group (**e**–**f**), the low-dose group (**g**–**h**), and the high-dose group (**i**–**j**). The *red arrowheads* show lysis of sarcolemma; the *yellow arrows* show chaotic arrangement of sparse myofibrils; the *green arrowheads* show swelling of mitochondria; the *blue arrowhead* show glycogen deposits

Examination of the ultrastructure of cardiomyocytes by transmission electron microscopy revealed that cardiomyocytes from the normal control rats (Fig. 5a–b) maintained sarcolemma integrity and orderly arrangement of myofibrils with sarcomere structures (i.e., Z lines and H zones) clearly seen. Elliptic mitochondria were linearly arranged without crista defects, swelling or vacuolation. No contracture zones or electron-dense deposits were detected. In contrast, cardiomyocytes from the DCM control and supernatant-treated rats (Fig. 5c–f) showed lysis of sarcolemma, chaotic arrangement of sparse myofibrils with local myofilament ruptures, and obscure sarcomere structures. Also observed were swelling and deformation of mitochondria with vacuolar degeneration and obscure crista structures. Glycogen deposits were present between myofibrils in some regions. On the other hand, cardiomyocytes from the low-dose (Fig. 5g–h) and high-dose (Fig. 5i–j) groups largely maintained sarcolemma and fascicle integrity, orderly arranged myofibrils, and clear sarcomere structures. Most mitochondria maintained their elliptic shape and some were arranged in a linear fashion. While mild swelling was present, crista structures were mostly uncompromised without vacuolar degeneration.

## Discussion

In the recent years, stem cells which promote tissue repair and angiogenesis [6] have emerged as a promising alternative treatment for fatal pediatric DCM [7]. However, the inherent invasive nature of intracoronary and intramyocardial administration [10] has significantly restricted their extensive clinical applications. In the present study, we have shown that intramuscular injection of UCMSCs induced significant improvement in cardiac function and attenuation in cardiomyocyte impairments in a DCM rat model. In addition, UCMSC treatment altered the expression of cytokines relevant to tissue repair and angiogenesis such as HGF, VEGF, and IGF-1 in the heart, suggesting that the therapeutic effects of intramuscularly administered UCMSCs might be mediated by paracrinal mechanisms.

MSCs are widely present in a number of tissues such as bone marrow, umbilical cord, adipose tissue, and the liver [16]. Previous studies have shown that intradermal injection of UCMSCs did not trigger allergic reactions [17], suggesting the low immunogenic capacity of UCMSCs. In the present study, intramuscular injection of hUCMSCs did not lead to local inflammation, ulceration or effusion in the injection sites. In addition, our preliminary experiments showed that intramuscular injection of hUCMSCs in normal rats did not induce alterations in heart, liver or kidney function (data not shown). These results indicate that intramuscular injection of hUCMSCs as a safe administration procedure.

The doxorubicin-induced DCM rat model used in this study developed ventricular enlargement associated with impaired contractility and elevation in BNP and cTNI, consistent with the clinicopathological profiles of DCM. Echocardiography examination showed that intramuscular injection of hUCMSCs improved the myocardial contractility of the DCM rats as evidenced by elevated LVEF and LVFS. In addition, the markers for cardiac function (BNP and cTNI) were decreased in the DCM rats after hUCMSC treatments. These results suggest that intramuscular injection of hUCMSCs improved cardiac function of DCM and attenuated DCM-triggered heart failure. While intramyocardial injection of UCMSCs at similar dosages has been reported to promote tissue repair for heart attack-induced damage [18, 19], the current study using intramuscular injection has provided by far a more preferred choice due to its minimum invasiveness.

However, treatment with hUCMSCs did not result in apparent changes in left ventricular dimensions within the time frame of this study. Nonetheless, examination of cardiomyocyte ultrastructure revealed that hUCMSC injections attenuated DCM-induced cellular impairments such as defected sarcolemma integrity, myofibril disorganization, and mitochondria degeneration, thus providing the structural basis at cellular level for hUCMSC-induced functional improvements. More injections and longer observation time might be needed for dimensional changes in the left ventricle to occur.

Consistent with the results of this study, previous studies by Shabbir et al [14] reported that intramuscular injection of similar quantities (at 10^6^ cells) of bone marrow-derived MSCs (BMMSCs) improved the cardiac function in a TO-2 DCM hamster model. However, intramuscular injection of conditioned medium induced significant improvement in ventricular dimensions and function in the TO-2 hamsters [14], which was not observed in the present study. The difference in pathogenesis between doxorubicin-induced DCM and genetic defects-induced DCM might be the underlying reason.

While the mechanisms underlying the therapeutic effects of MSCs on DCM remain poorly understood currently, it is generally believed that stem cells might achieve their therapeutic effects via paracrine mechanisms [20]. MSCs secrete a large number of bioactive molecules, including cytokines, antioxidants, pro-angiogenic molecules, and trophic factors, which can promote tissue repair via multiple pathways [6]. Nagaya et al [21] reported that BMMSCs injected into the myocardium of an autoimmune myocarditis rat model secreted a large amount of cytokines such as VEGF and IGF. In the present study, we have shown that at the low- and high-dose hUCMSC injections induced significant elevations in serum HGF, LIF, GM-CSF, and VEGF. Furthermore, the low- and high-dose hUCMSC treatments significantly increased the mRNA of HGF, VEGF, and IGF-1 in the myocardium, indicating the upregulation of the expression of those cytokines. All these cytokines are involved in cell proliferation, angiogenesis, tissue repair, and regeneration. VEGF protein was increased only in the myocardium of the high-dose treatment group, suggesting that VEGF may contribute to MSCs-induced tissue repair but does not act as a primary factor. Elevated secretion of these cytokines was observed in the DCM rat model before hUCMSC treatment, which might represent a spontaneous reaction of the body to repair the injured myocardium in response to the detrimental effects of doxorubicin. Further elevated expression and secretion of these cytokines after hUCMSC injections represent increased repairing capacity of the injured myocardium. Therefore, promoting the expression and secretion of tissue repair-related cytokines instead of forming myocardium by differentiation is likely to be the mechanism underlying the therapeutic effects of hUCMSCs administered via intramuscular injection. Actually, MSCs administered via intramuscular injection predominantly remain in the skeletal muscle rather than migrate to other organs [21].

As for the lack of effect by the conditioned medium, it is possible that continuous paracrine stimulation by hUCMSCs rather than one single dose of cytokines might be necessary to induce adequate changes in cardiomyocytes for cardiac function improvement.

By exploiting the paracrine effects of MSCs, intramuscular injection of MSCs has averted the risks involved in intracoronary and intramyocardial injections, thus providing a promising therapeutic strategy for pediatric DCM. Further studies are needed regarding the optimal timing of treatment, the optimal dosage of MSCs, the frequency of injections, and long-term prognosis.

## Conclusions

This study has provided evidence that intramuscular injection of hUCMSCs in DCM rats can improve cardiac function and attenuate cardiomyocyte damage by regulating the expression and secretion of multiple tissue repair-related cytokines via paracrine mechanisms. Therefore, intramuscular injection of MSCs may be a promising candidate treatment for pediatric DCM as a minimally invasive approach.

## Additional file

Additional file 1: Table S1. Alterations in serum BNP and cTNI levels by doxorubicin treatment. **Figure S1.** Development of DCM examined by echocardiography. **Figure S2.** Survival rate of DCM rats during doxorubicin treatment. **Figure S3.** hUCMSCs examined for growth capacity, differentiation capacity and immune phenotype. **Figure S4.** Cardiac fibrosis evaluated by Masson staining (DOC 5053 kb)

## Abbreviations

BMMSCs: Bone marrow-derived mesenchymal stem cells
BNP: Brain natriuretic peptide
CMV: Cytomegalovirus
cTNI: Troponin I
DCM: Dilated cardiomyopathy
GM-CSF: Granulocyte-macrophage colony-stimulating factor
HBV: Hepatitis B virus
HCV: Hepatitis C virus
HGF: Hepatocyte growth factor
HIV: Human immunodeficiency virus
IGF-1: Insulin-like growth factor-1
IVSD: Interventricular septal end-diastolic diameter
LIF: Leukocyte inhibitory factor
LVEF: Left ventricular ejection fraction
LVESd: Left ventricular end-systolic diameter
LVFS: Left ventricular fraction shortening
LVIDd: Left ventricular inner end-diastolic diameter
LVPWd: Left ventricular posterior wall end-diastolic diameter
MSCs: Mesenchymal stem cells
UCMSCs: Umbilical cord-derived mesenchymal stem cells
VEGF: Vascular endothelial growth factor
