# Supplementary material for: Intramuscular injection of human umbilical cord-derived mesenchymal stem cells improves cardiac function in dilated cardiomyopathy rats
Source: Stem Cell Res Ther. 2017 Jan 28;8:18. doi: 10.1186/s13287-017-0472-y (PMC5273808; doi:10.1186/s13287-017-0472-y)
Supplement: Additional file 1: Table S1. — Alterations in serum BNP and cTNI levels by doxorubicin treatment. Figure S1. Development of DCM examined by echocardiography. Figure S2. Survival rate of DCM rats during doxorubicin treatment. Figure S3. hUCMSCs examined for growth capacity, differentiation capacity and immune phenotype. Figure S4. Cardiac fibrosis evaluated by Masson staining (DOC 5053 kb) [file 13287_2017_472_MOESM1_ESM.doc]

**Additional file**

*Induction of DCM by doxorubicin injection*

The DCM rat model was established by intraperitoneal injections of doxorubicin (see details in the Methods and Materials). At the end of the procedure, echocardiography and peripheral blood BNP and cTNI were examined to assess heart dimension and function (Suppl. Fig.1 and Suppl. Table 1). Doxorubicin injection induced significant enlargement of the left ventricle as evidenced by increased LVIDd and LVESd accompanied by reduced LVPWd and IVST. Left ventricular systolic function was impaired as evidenced by decreased LVEF and LVFS. Furthermore, doxorubicin induced significant elevation in BNP and cTNI levels, suggesting compromised cardiomyocyte functions and development of heart failure.


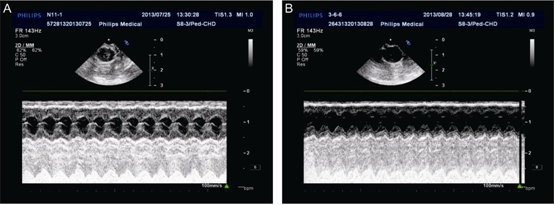


**Figure S1.** Echocardiograms of a normal control rat (A) and a DCM rat (B)

**Table S1. Doxorubicin injection on heart dimension and function**

|  | LVIDd  (mm) | LVESd  (mm) | LVPWd  (mm) | IVSD  (mm) | LVEF  (%) | LVFS  (%) | BNP  (pg/ml) | cTNI  (ng/L) |
| --- | --- | --- | --- | --- | --- | --- | --- | --- |
| Normal control | 6.2 ± 0.3 | 3.4 ± 0.1 | 2.3 ± 0.2 | 2.4 ± 0.2 | 79.7 ± 3.0 | 43.1 ± 3.2 | 221 ± 55 | 107 ± 37 |
| DCM | **4.5 ± 0.5** | **3.9 ± 0.2** | **1.6 ± 0.4** | **1.9 ± 0.4** | **64.5 ± 2.6** | **30.8 ± 2.1** | **450 ± 92** | **426 ± 43** |

* Boldface represents statistical difference (P < 0.05).

*Doxorubicin injection on survival of the rats*

Rats in the DCM group started to die from the fourth week after the initiation of doxorubicin injection, and at two week after the end of the procedure (i.e. the time when hUCMSC treatment commenced), 82 out of the 140 (58.6%) rats survived. Autopsy revealed hepatomegaly, hepatonecrosis, sanguineous ascites, pleural effusion, renomegaly and intestinal obstruction. Suppl. Fig.2 showed the survival profile of the DCM rats along the time line.


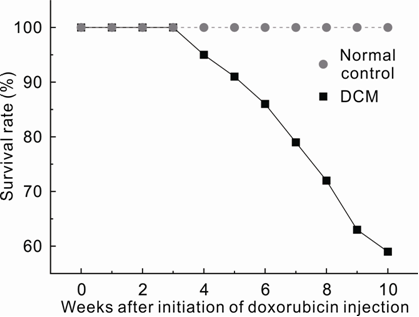


**Figure S2.** Survival profile of the rats along the time line of DCM induction

*Quality control of hUCMSCs*

The hUCMSC preparations (P#2) were tested for self-renewal capacity by monitoring their proliferation (Suppl. Fig.3A). To examine their differentiation capacity, the cells were seeded in 12-well plates at 1 × 10^4^ cells/cm^3^ and cultured in either an osteogenic induction medium (DMEM, 10% fetal bovine serum, 4 mM glutamine, 100 nM dexamethasone, 10 mM sodium β-glycerophosphate, 50 μM ascorbic acid-2-phosphate and 10 ng/ml bone morphogenetic protein 2) or an adipogenic induction medium (DMEM, 10% fetal bovine serum, 4 mM glutamine, 0.5 mM isobutyl-methylxanthine, 1 μM dexamethasone, 10 μM insulin and 200 μM indomethacin). The medium was replaced every three days, and after 14 days, osteogenic and adipogenic differentiation was examined by staining with alkaline phosphatase and Oil Red O respectively (Suppl. Fig.3B). Furthermore, the immune phenotype of the cells was determined by flow cytometry using a BD FACSCalibur flow Cytometer (BD Biosciences, San Jose, CA). The UCMSC identity of the cells was verified by immune phenotyping as CD34^−^/CD45^−^/HLA-DR^−^ and CD44^+^/CD90^+^/CD105^+^ (Suppl. Fig.3C).


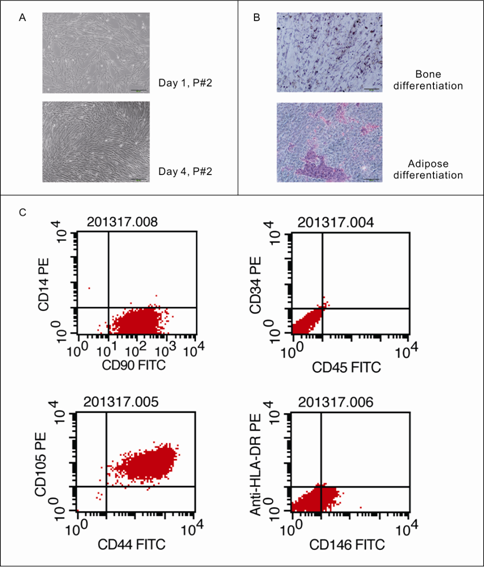


**Figure S3.** Quality control of the hUCMSC preparations

*Cardiac fibrosis evaluated by Masson staining*


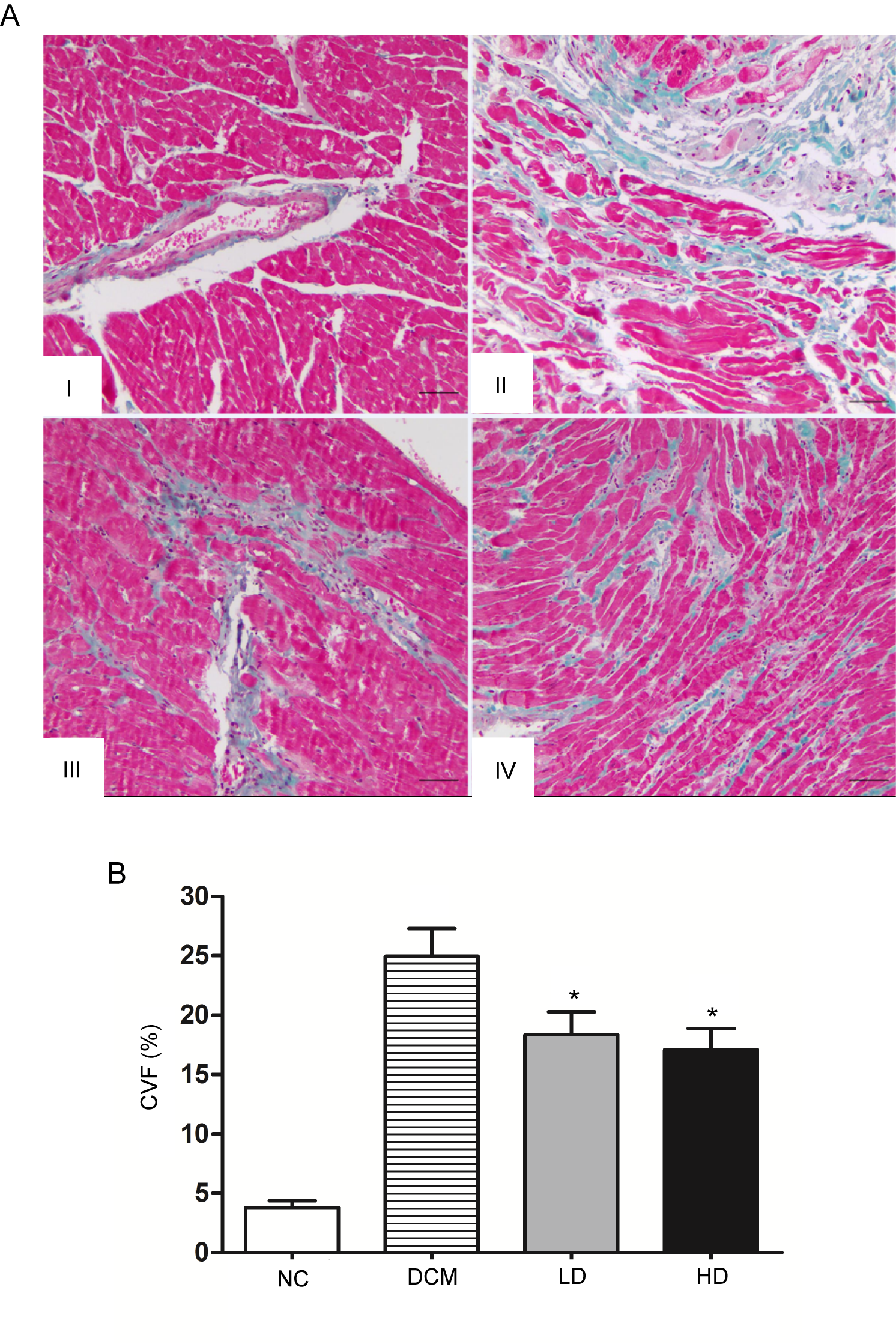


**Figure S4.** Masson staining of cardiac tissue **(A).** (I) the Normal group. (II) the DCM control.(III) the Low-dose group. (IV) the High-dose group. Green represents collagen fiber in the figure. Black bars = 50 μm. **(B)** Statistic analysis of CVF. Asterisks represent statistical difference compared with DCM group (*P* < 0.05, ANOVA).
